# Finite Element Simulation and Optimization of Process Parameters for Titanium Chip Crusher

**DOI:** 10.3390/ma18091894

**Published:** 2025-04-22

**Authors:** Jianghua Huang, Zeling Zhao, Xiaomin Huang, Tao Liu, Hongchao Ji

**Affiliations:** 1 School of Mechanical and Energy Engineering, Zhejiang University of Science and Technology, Hangzhou 310023, China; huangjianghua@zust.edu.cn; 2 College of Mechanical & Energy Engineering, Beijing University of Technology, Beijing 100124, China; 3 College of Mechanical Engineering, North China University of Science and Technology, Tangshan 063210, China; xmhuang@ncst.edu.cn; 4 China MCC 22 Group Corporation Ltd., Tangshan 063035, China; mccliutao@163.com

**Keywords:** TC4, titanium chips, Johnson–Cook constitutive model, double-teeth roll crushing process, finite element simulation

## Abstract

Titanium alloy has been widely used in aerospace, military, and national defense, and other high-tech fields due to its advantages of light weight, high specific strength, excellent corrosion resistance, excellent heat resistance, and good low-temperature performance. In the turning of titanium alloys, a significant quantity of continuous chips with poor breakability is generated. Recycling these titanium chips can effectively reduce raw material costs and minimize environmental impacts, as it decreases the dependency on primary titanium sources. The titanium chip crushing process is an indispensable part of the titanium chip recovery process, and the double-teeth roll crushing process is the most commonly used metal crushing process. Finite element simulation is an efficient, time-saving, and resource-saving method to optimize the titanium chip crushing process. In this paper, the stress change in the process of titanium chip crushing is analyzed by finite element simulation, and the influence of the number of cutter roller teeth and the speed of the cutter roller on the crushing effect of the titanium chip is analyzed. The optimal process parameters of the titanium chip crusher were obtained by response surface optimization, and the accuracy of the finite element simulation was verified by experiments, which provided a theoretical and practical basis for optimizing the titanium chip crushing process.

## 1. Introduction

With the development of science and technology, the requirements for materials, especially metal materials, are increasing in various industrial fields. Titanium alloy is widely used in aerospace, military defense, and other high-tech fields because of its light mass, high specific strength, good corrosion resistance, high thermal strength, and good low-temperature performance [1,2,3]. The main components of titanium alloy are α-phase, β-phase, and α + β phase titanium alloy, and the two-phase titanium alloy is most widely used. The α + β phase titanium alloy has good comprehensive performance and a stable structure. TC4 is a typical two-phase titanium alloy [4,5,6]. Due to the unique processing characteristics of a titanium alloy, a large number of chips will be produced in the turning process. How to recycle these titanium alloy chips and reduce the costs of titanium production has become an important research direction in the titanium processing industry.

The metal crushing process is an indispensable part of the metal recovery process. Double-tooth roller crushers have been widely used because of their simple structure, reliable work, and low costs [7]. However, most of the twin-tooth roller crushers are developed for ore, and there are few studies on ductile metal crushing processes with low hardness such as titanium chips [8,9,10,11]. Ordinary ore crusher work is to rely on the adjacent two teeth of the ore impact extrusion crushing, and the state of titanium chip crushing is different; titanium chip crushing needs to form a shear mouth between the teeth roller, using the shear force to break the titanium chip [12,13].

In previous times, numerous researchers have delved into the study of crushers and their associated crushing processes. André et al. [14] used a discrete element model to simulate the crushing of mixed materials containing hard and soft materials in a cone crusher. The results show that the crusher output will not decrease with the proportion of ductile materials. Neto et al. [15] developed a discrete-event simulation model to improve the performance of a steel production line and applied the model to a steel production line, showing that it could significantly improve the efficiency of the line. Kwon et al. [10] established a crushing-agglomeration combination model to characterize the particle characteristics of the double-teeth roller crusher and predict the particle size distribution of the crusher products. This model can represent the decrease and increase in the complex particle size in the process. The experimental parameters were used to predict the particle size distribution of the products with distributed particle size feed, and the results were compared to the experimental results, which were in good agreement. This indicates that this model can better predict the product size distribution. Lieberwirth et al. [16] studied the load variation law of the double-teethed roller crusher under extreme conditions. Considering the fact that a large amount of energy is stored in the flywheel and the mass of the floating roller of the maximum machine is too large, suggesting that any unexpected dynamic behavior may raise issues of safe operation, and obtained the crushing force dynamics results under extreme conditions, as well as the influence of the particle size distribution of related products and the mineral structure of various rocks on the clamping behavior. Marcus Johansson et al. [17] established the time dynamic response model of a high-pressure roller mill and studied the dynamic response of hydraulic system damping, feed particle size change, ore hardness, and crusher-to-roller speed change. The model is suitable for high-fidelity analysis and shows the potential of predicting the performance of a crusher in process simulation, with an average error of about 10%.

Finite element simulation is an effective way to study the titanium chip crushing process and can effectively reduce research costs through finite element simulation [18,19,20,21,22]. ABAQUS has a huge database and a powerful three-dimensional modeling function; it can also import the built model directly into the software. Its rich material attribute table can directly give the model material and analyze its properties [23].

A reasonable intrinsic constitutive model is a prerequisite for accurate finite element simulation results. Over the years, many researchers have investigated the deformation properties of materials under different loading conditions and modified many constitutive models to describe the flow behavior. These models can be divided into the following three categories: empirical model, semi-empirical model, and physical-based model [24,25,26]. For example, the Johnson–Cook model, the Zerilli-Armstrong model, the Steinberg-Guinan model, and the Cowper-Symonds model [27,28,29,30]. Among them, the Johnson–Cook constitutive model has been widely used because it involves fewer material constants and can be directly applied to a variety of finite element software, especially for predicting the mechanical properties of materials under different conditions [31,32,33,34].

In this paper, SolidWorks 2017 software is used to model the double-tooth roller crusher, and a multi-group crushing tooth roller model based on different tooth numbers is established. Then the model is imported into ABAQUS for simulation. The effects of different numbers of cutter teeth, different rotational speeds, and different tip angles on the crushing of titanium chips were considered. Through finite element simulation, the stress change during the crushing process of titanium chips was analyzed, and the influence of the number of cutter roller teeth and the speed of the cutter roller on the crushing effect of titanium chips was analyzed. The optimal process parameters of the titanium chip crusher were obtained by response surface optimization, and the accuracy of the finite element simulation was verified by experiments, which provided a theoretical and practical basis for optimizing the titanium chip crushing process.

## 2. Model and Simulation of Titanium Chip Crushing Process

### 2.1. Selection of Cutter Tooth Profile Structure of Double-Toothed Roll Crusher

When the double-toothed roll crusher is working, the installation of different types of crushing teeth will directly affect the crushing effect of the processed materials and the energy consumption during processing and will also affect the output of the crusher and the service life of the cutter teeth. Therefore, it is necessary to conduct a comprehensive analysis of the common broken tooth structure in the market. The crushing behavior of different cutter tooth structures on the material during operation is different, including shearing, extrusion, collision, and puncture. Considering the characteristics of low stiffness and good toughness of titanium chip material, a cutter tooth structure suitable for titanium chip crushing is designed.

**Figure 1:**
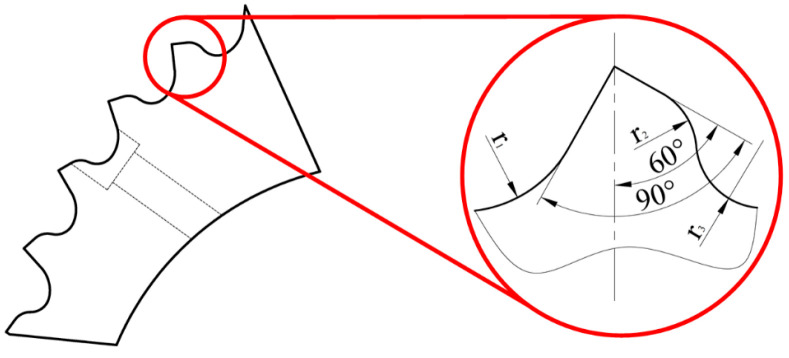
Schematic diagram of tooth plate and crusher tooth structure.

There are many factors affecting the design of the tooth structure of the double-toothed roll crusher, especially for the particle size distribution of the product and the material properties of the processed materials. Therefore, considering the material properties of titanium chips and the requirements for their processing granularity, a broken tooth with simple structure is proposed. Although it only involves the basic shape parameters of the broken tooth, it can generally describe the shape of the broken tooth and provide a good basis for further optimization design of the tooth shape. As shown in Figure 1, the tooth structure of the tooth plate crushing tooth for titanium chips is shown.

**Figure 2:**
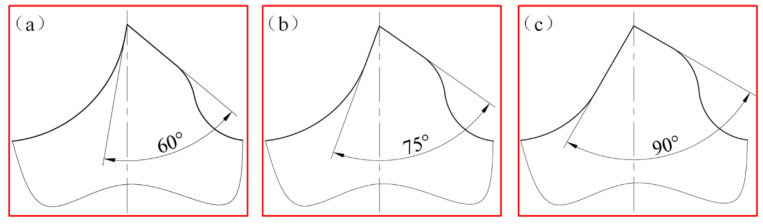
Schematic diagram of crusher teeth with different cutting edge angles. (**a**) 60° blade angle. (**b**) 75° blade angle. (**c**) 90° blade angle.

In order to study the influence of different cutter tooth shapes on the stress of titanium chips when the crusher is working, 60°, 75°, and 90° are selected as the cutter tip angles to model the cutter. Figure 2 is a schematic diagram of broken teeth with different tip angles. Among them, (a) corresponds to a cutting-edge angle of 60°, (b) to 75°, and (c) to 90°.

### 2.2. Model of Titanium Chip Crushing Teeth Roller

The establishment of a geometric model is the basis of finite element analysis, and the establishment of an accurate 3D model can improve the accuracy of simulation results [35]. Therefore, how to establish the 3D model efficiently and accurately is particularly critical. If the 3D model is too complex, it will bring considerable difficulties to the later finite element analysis. So, it is necessary to make a reasonable simplification of the 3D model of the titanium chip crushing roll.

**Figure 3:**
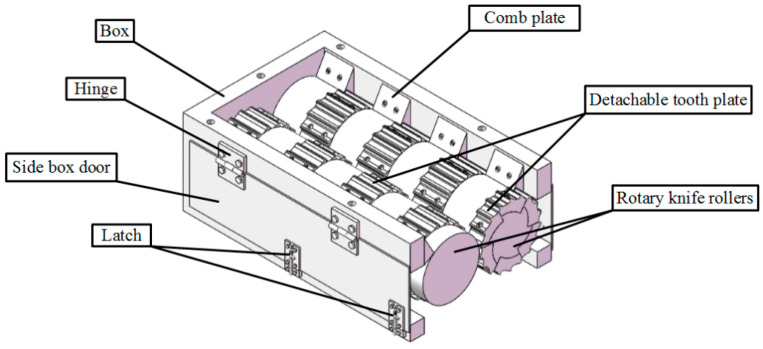
Double-teethed roller titanium chip crusher.

Figure 3 is the overall model of the double-teethed roll titanium sheet crusher. The broken teeth plate is fixed on the broken teeth roller by bolts. To facilitate the later finite element software to mesh the broken teeth roller and save the simulation calculation time, only a part of the broken teeth roller is taken to establish the simulation model.

To explore the influence of different tooth numbers on the crushing effect of titanium chips, SolidWorks 2017 software was used to model the crushing knife roller, and 18, 23, 27, and 32 tooth numbers were used for the model. Then it is imported into the finite element software ABAQUS 2020 for calculation.

To study the effect of different rotational speeds on titanium chip crushing, different simulation groups were established at different rotational speeds of 18 rpm, 22 rpm, 26 rpm, and 30 rpm.

### 2.3. Simulation of Titanium Chip Breaking Process

To greatly reduce the calculation time and amount of calculation of the titanium chips crushing simulation, the established model should be simplified as much as possible on the premise of improving the accuracy of simulation calculation results. So, to more effectively apply the finite element analysis of the titanium chip crushing process, in this paper, the following settings were used:

(1) The ambient temperature is always 25 °C.
(2) The cutter is a sharp, rigid body, and it only rotates.
(3) The processed work-piece material is isotropic.
(4) All the chemical changes due to temperature changes during the crushing of titanium chips are not considered.
(5) The titanium chip material transition temperature is set at 25 °C.

Since the hardness of the tool material is much higher than that of the titanium chip, the elastic modulus of the tool material is large compared to that of the titanium chip material. When the titanium chip is broken, the titanium chip will have large plastic deformation, and the deformation of the tool can be completely ignored. Therefore, in this paper, the tool is set as a rigid body [36].

The most important step in the process of establishing the finite element model is to divide the finite element mesh. Many factors should be considered when meshing because the calculation accuracy and calculation scale of the simulation model are limited by the number and shape of the mesh [37].

Usually, two specific parameters are used to determine the number of meshes. One parameter is the number of elements divided by the selected solid surface, and the other parameter is the mesh density control parameter. Through the trade-off between these two parameters, the accurate division of finite element meshes is finally achieved.

In finite element simulation analysis software, the commonly used mesh elements mainly include the following two types: mixed tetrahedral elements and eight-node hexahedral elements [38]. The mesh generation of mixed tetrahedral elements is relatively easy because its geometric characteristics are linear. However, the mesh division of eight-node hexahedral elements is more difficult, but it is suitable for deformation analysis and heat conduction analysis.

In the process of finite element simulation analysis, sometimes the analysis is terminated due to some extreme phenomena, which are mainly due to the serious deformation of the element mesh. This phenomenon will seriously affect the accuracy of simulation results and even lead to the termination of the analysis process. To solve this phenomenon, the grid should be re-divided, that is, the abnormal grid should be divided.

**Figure 4:**
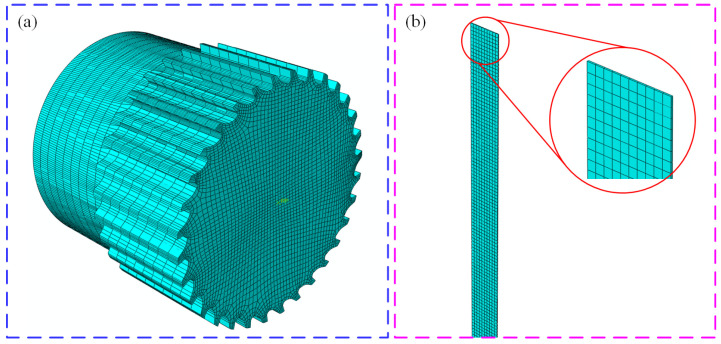
Meshing division for the knife roll and titanium chips. (**a**) Broken knife roller model (**b**) Titanium chip model.

Finite element software enables automatic mesh re-division to deal with mesh distortions [39]. Figure 4a shows the model of the rotating cutter roller after tetrahedral meshing by finite element software. The model is a rigid body, shell element. Figure 4b is the titanium chip model after meshing using a hexahedral mesh solid model.

A prerequisite for the accuracy of the crushing process in titanium chip crushing simulation is to obtain an accurate material model. The TC4 titanium alloy was selected as the titanium chips material. In finite element software, the following parameters need to be set: material density, elastic modulus, conductivity, Poisson’s ratio, inelastic heat share, expansion coefficient, specific heat capacity, Johnson–Cook damage coefficient, and Johnson–Cook constitutive parameters. See Table 1 and Table 2 for the properties of titanium chip materials. Among them, the Johnson–Cook constitutive model setting parameters can be obtained by reference [40].

Material density: t/mm^3^, elastic modulus: MPa, conductivity: W/(m·K), expansion coefficient: 1/°C, and specific heat capacity: J/(kg·°C).

The Johnson–Cook failure model parameters d1, d2, d3, d4, and d5 of TC4 titanium alloy can be obtained by referring to the literature [40]. Table 3 shows the Johnson–Cook damage parameters used in finite element software.

The built model is imported into finite element software, given titanium chip material, and the model is assembled. Through the interaction between the titanium chip and the crushing roller, the load was applied to the model, and the finite element model was established and solved.

## 3. Results and Discussion

### 3.1. Processing of Simulation Results

In the cutting process, when the tool is in contact with the titanium chip, the titanium chip first undergoes elastic deformation. With the rotation of the knife roller, the teeth on the knife roller continuously interact with the titanium chips for the shearing operation. With this continuous meshing and the shearing process, the accumulated stress on the titanium chips gradually increases, resulting in plastic deformation of the titanium chips. Finally, after the stress reaches a certain degree, the titanium chips break.

**Figure 5:**
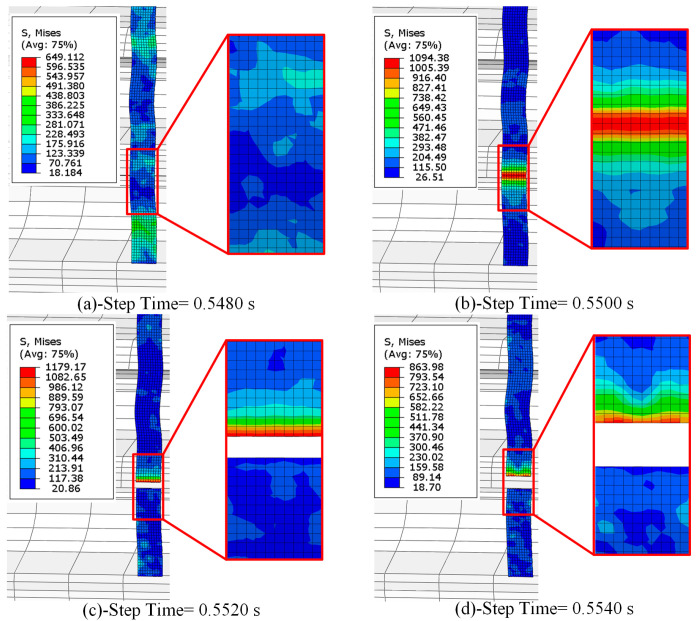
Stress cloud chart of the titanium chip fracture process. (**a**) elastic deformation. (**b**) plastic deformation. (**c**) break. (**d**) break.

The crushing process of titanium scraps can be divided into two stages. The first stage is the initial stage, during which the titanium scraps just begin to contact the crushing roller teeth, and the stress on the titanium scraps gradually increases. However, the contact position between the titanium chips and cutter teeth remains in the elastic deformation stage, with no yield or plastic deformation occurring, as illustrated in Figure 5a; With the continuous action of external load, the stress concentration at the contact position gradually increased, the titanium chip was continuously compressed, and yielding and plastic deformation occurred, as shown in Figure 5b. The second stage is the fracture stage of titanium chips. Under the continuous shear extrusion of the crushing roller, the titanium chips are sheared, and the plastic deformation zone is continuously thinning until the crushing teeth are fully engaged with another crushing roller. At this time, the titanium chips are completely fractured, and the stress at the fracture position of the titanium chips reaches the maximum, as shown in Figure 5c. The unit is continuously removed during the fracture of the titanium chips, and the two sections are gradually separated while the stress on the chip gradually becomes less, as shown in Figure 5d.

**Figure 6:**
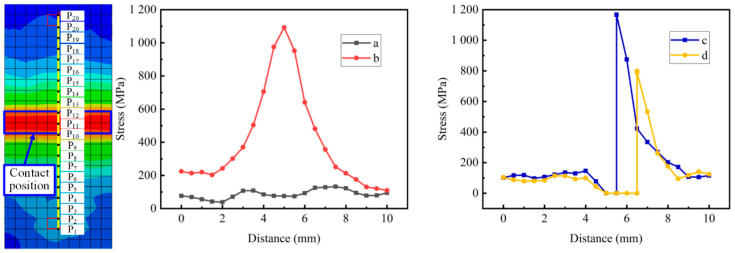
Distance–stress curve of the titanium chip fracture process.

Ten nodes were selected from each side of the center of the titanium chip fracture position, and the distance between each two nodes was set to 0.5 mm. According to these nodes, the respective stress values are calculated, and the point-line diagram is drawn (as shown in Figure 6). Figure 6 shows the stress distribution of the nodes at the fracture of titanium chips in the four stages of a-d in Figure 5. It can be seen from the point-line diagram that when the cutter teeth contacted the titanium chip at the beginning, the stress fluctuation of the titanium chip was small. With the continuous effect of external load, when the plastic deformation of the titanium chip began to occur, as shown in Figure 5b, the stress of the contact point increased rapidly, and the stress on both sides of the central point decreased with the increase in distance. It can be seen from curves c and d that when the titanium chip is broken, the stress of the cut titanium chip decreases. At the initial fracture time, the remaining titanium chip exhibits a stress peak. The maximum stress on the titanium chips decreases over time, and the stress on each node diminishes with increasing distance.

**Figure 7:**
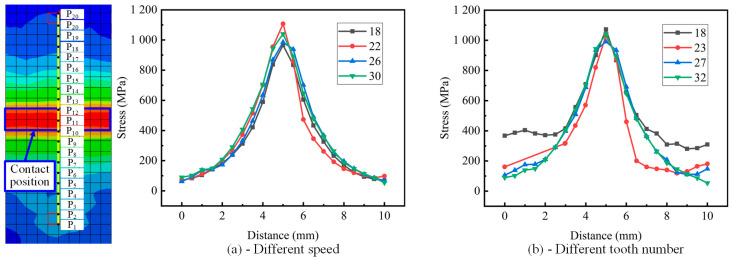
Stress distribution of titanium chips under different tooth numbers and rotational speeds. (**a**) Different speed. (**b**) Different number of teeth.

The simulation experiment was carried out on the titanium chip crushing process under different tooth numbers of cutter rolls and different crushing roll speeds. At the same moment (the initial moment of plastic deformation), 10 nodes were selected from the contact position of the cutter teeth and the titanium chip to both sides, and the stress of each node was extracted. The distance–stress curve is shown in Figure 7. It is found from the figure that the distance–stress curves under different tooth numbers of cutter rolls are very similar. It is found from the curve that when the plastic deformation of the titanium chip begins, the stress at the contact position between the titanium chip and a cutter tooth increases, and the stress at each node decreases with the increase in distance.

It can be seen from Figure 7b that the stress fluctuation of titanium chips on both sides of the contact position of different tooth numbers is larger. The stress change in titanium chips increases with the increase in teeth number, and the curve changing to both sides is smoother, and the stress change is more stable.

**Figure 8:**
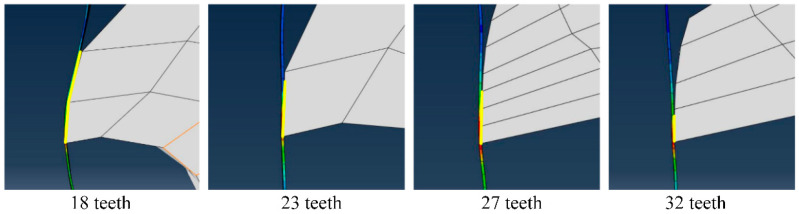
Contact position of titanium chip and roller teeth.

The four figures in Figure 8 correspond to the four curves in Figure 7b. It can be seen that due to the different number of teeth, the teeth profile structure is also different, which leads to the different contact areas between the teeth roller and the titanium chip. Due to the large angle of the blade, some tooth rollers not only contact with the titanium chips at the tip of the tooth, but also contact with the titanium chips at other positions. This means that in addition to the force at the tip of the tooth, the titanium chip will also be subjected to the force of other parts, resulting in a large stress fluctuation on both sides of the contact point.

**Figure 9:**
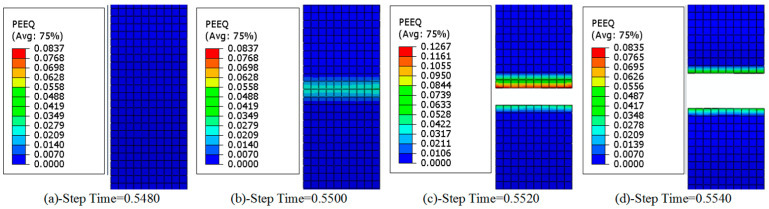
Equivalent plastic strain during titanium chip breaking. (**a**) elastic deformation. (**b**) plastic deformation. (**c**) break. (**d**) break.

As shown in Figure 9, the equivalent plastic strain cloud chart of the titanium chip crushing process at 32 teeth and a rotating speed of 30 rpm is shown. Figure 9a–d correspond to Figure 5a–d, respectively. It can be seen from Figure 9a that when Step Time = 0.548, there is no change in the strain cloud chart. This is because the initial contact between the titanium chip and the crushing roller teeth is at this time. The stress change in the titanium chip at this time is small. At this time, the titanium chip is in the elastic change stage, and no plastic strain occurs.

Figure 9b under the shearing action of crushing roller teeth, plastic deformation of titanium chips begins due to the increase in stress. Figure 9c shows that the titanium chip has been broken and fractured at this time. The plastic deformation at the fracture site is the largest, and the degree of plastic deformation decreases with the distance to both sides. After the crushing, the stress of the titanium chip is gradually smaller, and the plastic deformation is also reduced, as shown in Figure 9d.

**Figure 10:**
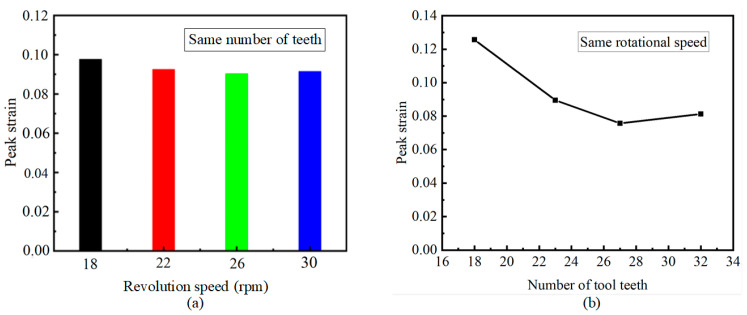
Peak statistics of equivalent plastic strain under different conditions. (**a**) The peak value of equivalent plastic strain at different rotational speeds with the same number of teeth. (**b**) The peak value of equivalent plastic strain under the same rotational speed and different number of teeth.

Figure 10a shows the peak value statistics of the equivalent plastic strain of the titanium chip in the process of the titanium chip crushing under different rotational speeds with the same number of teeth (32 teeth). It can be seen that the peak strain difference at different speeds is small and there is no obvious fluctuation. Figure 10b shows the peak value statistics of the equivalent plastic strain of the titanium chip in the process of the titanium chip crushing under the same rotational speed (30 rpm) and different tooth numbers. It can be seen that when the tooth number of the crushing roller is 18, the peak strain is the largest, followed by 20 to 32.

Four simulation models are established with 18 teeth, 23 teeth, 27 teeth, and 32 teeth at the same rotational speed. The broken models are measured and counted, and the results are shown in Table 4.

Through the simulation of the titanium chip crushing process, it is found that the initial contact position between the titanium chip and the crushing roller is different in the four groups. Thus, shorter titanium chips were obtained, respectively, 0.5 mm, 2.4 mm, 5 mm, and 7 mm.

**Figure 11:**
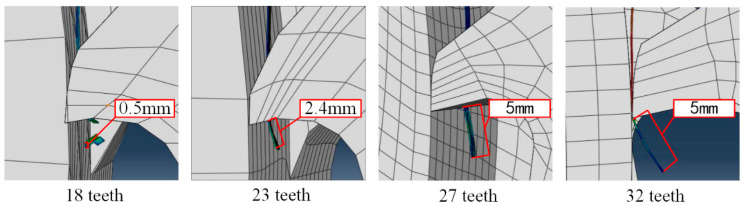
Initial contact position between the tool and a titanium chip.

As shown in Figure 11, the contact position between the initial tool and the titanium chip is found. It is found that the length of such titanium chips varies greatly, so it cannot be included in the analysis of the crushing effect of titanium chips.

Under the condition of the same tooth number, the simulation analysis of different crushing roller speed crushing models is carried out. Statistical analysis of the simulation results is shown in Table 5, Table 6, Table 7 and Table 8.

It is also found that due to the different initial contact positions between the tool and the titanium chip, the shorter titanium chips are produced at the beginning, and similarly, shorter titanium chips are produced after the titanium chip is broken. The crushing length of such titanium chips is not stable, which cannot be used as a basis for analyzing the crushing effect of titanium chips.

### 3.2. Effect of Different Model Parameters on Titanium Chip Crushing

**Figure 12:**
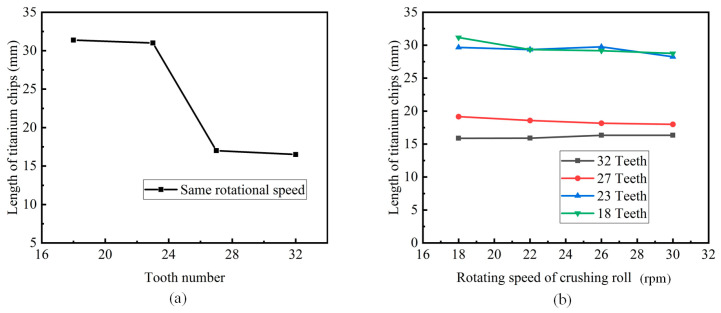
The influence of tooth number and rotational speed on the crushing effect of titanium chips. (**a**) The influence of tooth number on the crushing effect of titanium chips. (**b**) The effect of rotational speed on the crushing effect of titanium chips.

The above simulation results were processed to remove the obvious deviation results, and the remaining values were averaged, and the point-line diagram was drawn. Figure 12a and Figure 12b, respectively, show the influences of different tooth numbers and different crushing roll speeds on the titanium chip crushing effect.

It can be seen from Figure 12a that with the increase in the number of crushing roller teeth, the crushing effect of titanium chips gradually becomes better, and the length of titanium chips after the crushing becomes shorter. It can obviously be found that with the increase in the number of teeth, the length of titanium chips after crushing changes more obviously. When the model was established, it was assumed that the top circle and the teeth and root circle of all crushing rollers were identical. Consequently, as the number of teeth increases, the distance between the adjacent teeth decreases. This implies that the length of crushed titanium chips diminishes with a reduced tooth distance, resulting in a more effective crushing effect.

It can be considered that the length of crushed titanium chips decreases as the distance between the two teeth decreases and the crushing effect becomes increasingly effective.

As can be seen from Figure 12b, when the number of crushing roller teeth is constant, the speed of the crushing roller has negligible impact on the length of titanium chips after crushing. When the rotational speed varies for each tooth number model, the length of the crushed titanium chips does not change significantly, suggesting that the rotational speed has little influence on the crushing effect of titanium chips.

## 4. Parameter Optimization of Double-Teethed Roll Crusher Based on Response Surface Method

In the process of titanium scrap crushing production, there are many factors that affect the crushing effect of titanium scraps. Among them, the number of teeth of the rotating cutter roller, the rotation speed of the cutter roller during the processing, and the angle of the tip of the broken teeth are directly related to the crushing effect of titanium scraps. The response surface optimization test is combined with the finite element simulation test. The number of teeth of the cutter roller, the speed of the cutter roller, and the angle of the tool tip are the influencing factors of the test. The average value of the broken length of the titanium scraps after crushing is used as the response value. By establishing the regression curve between the test factors and the response value, the influence of the test factors on the response value was analyzed.

The BBD experimental design method was used in this titanium chip crushing test. There are three factor values, and the actual values are presented in the design table. The experimental design scheme is shown in Table 9. In the test table, the fourth group, the sixth group, the tenth group, the fourteenth group, and the fifteenth group were the central group. To reduce the test error, the central group was repeated five times. In this response surface test, the number of teeth of the crusher was 22 to 30, the speed of the crushing teeth roller was 22 rpm to 30 rpm, and the angle between the crushing teeth was 60° to 90 °. Based on these, the finite element simulation test parameters of the double-teethed roller crusher are determined as shown in Table 9.

The simulation calculation is carried out according to the test scheme. The Box-Behnken response surface quadratic nonlinear model was established, and the variance analysis and significance test were carried out. The model determination coefficient is R2 = 0.9915, and the coefficient of variation is C.V = 2.88%. Table 10 shows the variance analysis of the crushing length of titanium chips. P and F are two important measurement parameters in the table. The *p* value is an index to measure the difference between the control group and the experimental group. When the *p* value is less than 0.1, the difference between the two groups is very significant. The larger the F value, the higher the significance of the selected model. From Table 10, one can see that the F value of the selected quadratic nonlinear model is 35.29. According to the previous description, the larger the F value, the more significant the model is. According to *p* < 0.0001, the probability that the accuracy of the model is affected by error is low. The F value of the first term A is 148.78, and the *p* value is less than 0.0001, indicating that the number of teeth has a significant effect on the response value.

The regression equation with the average length of titanium chips after crushing as the response index is obtained by fitting:

(1) $$\begin{array}{l} f=0.2102A^{2}-0.0117B^{2}+0.0009C^{2}+0.0172AB-0.0020AC-\\ 0.0004BC-12.5187A+0.1500B-0.0883C+200.2125 \end{array}$$

**Figure 13:**
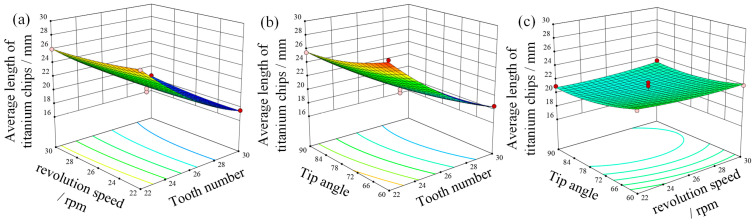
Response surface diagram of the average length of titanium chips after crushing. (**a**) Rotation speed and number of teeth. (**b**) Angle and number of teeth. (**c**) Angle and rotation speed.

Figure 13 shows the response surface model of the mean length of titanium chips after the crushing. From the figure, it can be seen that the number of teeth of the crushing roller is the most significant factor affecting the crushing length of titanium chips, among all the design parameters, and the length of titanium chips after crushing decreases with the increase in the number of teeth, but the length of titanium chips is not sensitive to the change in the speed of the crushing roller and the angle of the tool tip.

Taking the length of broken titanium chips as 20 mm as the optimization goal, the obtained regression equation is optimized. The mathematical model established according to the actual situation is as follows:

(2) $$Y=\min f\left(A,B,C\right)$$

After the calculation, the optimal solution of each parameter is *A* = 27.25, *B* = 22 rpm, and *C* = 90°; since the parameter *A* is the number of crushing teeth of the crusher, it must be an integer, so the optimal parameter *A* = 26 of the crusher is selected by the nearest principle. The optimized design parameters of the crusher were used to model the crushing teeth roller of the double-teethed roller crusher, and the finite element simulation was carried out. The average value of the simulation results was statistically averaged. The average length of the titanium chips after the crushing was 22.88 mm, and the error with the response surface optimization target was 0.055, which further verified the accuracy of the optimization results.

## 5. Experiment

**Figure 14:**
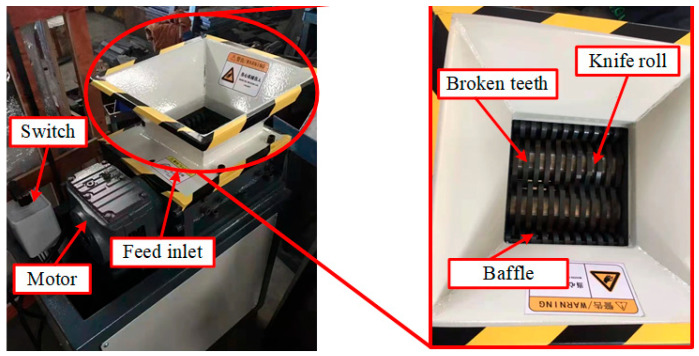
Double-tooth roller crusher for experiment.

Figure 14 shows a small double-tooth roller crusher with 26 teeth, including a motor, switch, feed inlet, knife roll, and comb plate. The TC4 titanium alloy chips were crushed by double teeth roller crusher, and the working speed of the crushing test machine was 18 rpm.

**Figure 15:**
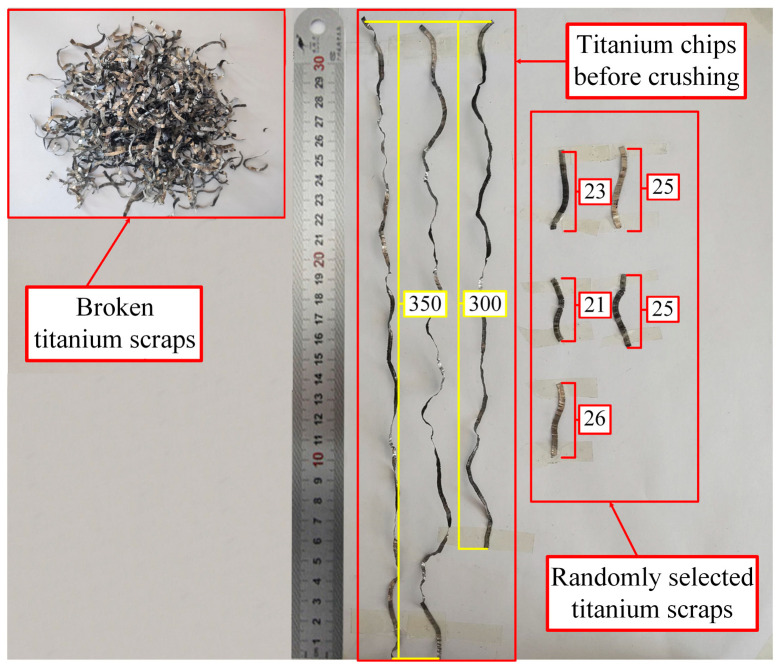
Length comparison of titanium chips before and after crushing.

Figure 15 shows the broken titanium chips. The test results show that titanium chips can break well.

Figure 15 presents the length comparison of titanium chips before and after the crushing process. As illustrated in the figure, the length of titanium chips prior to crushing surpasses 300 mm, with some reaching approximately 350 mm. Conversely, the length of the crushed titanium chips is about 25 mm. The experimental results are in excellent accordance with the finite element simulation results, thereby providing a solid foundation for the simulation.

## 6. Conclusions

Taking the TC4 titanium alloy ribbon-cutting chips (titanium chips) as the processing object, the double-tooth roller crusher model was established. Through the finite element crushing simulation, the influence of different broken roller tooth numbers and different broken roller speeds on the titanium chip crushing effect was systematically studied. The following conclusions can be drawn:

(1) Through the finite element simulation experiment, the stress change in the process of titanium chip breaking is obtained, and the stress change cloud diagram of the titanium chip from the initial contact position to the end of complete fracture is obtained. The stresses on the titanium chips in the simulation experiments with different numbers of teeth and different speeds were compared and analyzed, and it was found that as the number of teeth increased, the stresses on the chips changed more smoothly towards the sides and the stresses changed more stably.
(2) The influence of the number of crushing roller teeth and the speed of the crushing roller on the crushing effect of titanium chips was predicted by finite element simulation. The results show that with the increase in the number of teeth of the crushing roller cutter, the length of the broken titanium chip decreases gradually, and with the increase in the number of teeth, the crushing effect is more obvious. With the increase in the rotational speed of the knife roller, the processing speed of titanium chips becomes higher. That is, the number of cutter teeth has great influence on the length (particle size) of the titanium chip after the crushing, and the rotational speed of the cutter roller has little influence on the crushing particle size but has great influence on the working efficiency of titanium chip crushing.
(3) With the production of titanium chip crushing and the particle size of titanium chip after the crushing as the processing objectives, the number of cutter teeth and the rotational speed of the cutter roller can be adjusted to select the appropriate tool parameters and then provide the processing efficiency.
(4) The experimental design for optimizing the design parameters of the double-tooth roller crusher using response surface methodology revealed that the number of crushing roller teeth is the most significant factor affecting the titanium chip fragmentation length among all design parameters. Furthermore, the length of the crushed titanium chips decreases with an increase in the number of teeth, while the chip length is insensitive to changes in the crusher roller speed and cutting edge angle. The optimal design parameters for the crusher were determined to be 27 teeth for the crushing roller, a roller speed of 22 r/min, and a cutting edge angle of 90°.

## Figures and Tables

**Table 1:** Johnson–Cook model setting parameters.

Parameter	*A* (MPa)	*B* (MPa)	*n*	*C*	*m*	Melt Temperature (°C)	Transition Temperature (°C)
TC4	1003.132	1003.510	0.663	0.0137	1	1605	25

**Table 2:** Property parameters of TC4 titanium alloy material.

Parameter	Density	Elastic Modulus	Conductivity	Poisson’s Ratio	Inelastic Heat Share	Expansion Coefficient	Specific Heat Capacity
TC4	4.43 × 10^−9^	108,000	6.8	0.33	0.9	9.0 × 10^−6^	520,000

**Table 3:** Johnson–Cook damage parameters.

d1	d2	d3	d4	d5	Melting Point °C	Transition Temperature °C	Reference Strain Rate
−0.197	2.332	3.138	0.034	3.87	1605	25	0.001

**Table 4:** Finite element prediction of the effect of different numbers of crushing teeth on the crushing effect of titanium chips.

Teeth Number	Length of Titanium Chip After Breaking/mm					
18	0.5	18	19.5	39	49	-
20	2.4	7	28.5	33.5	-	-
27	5	7	12	17	19	20
32	5	12	18	19	23.5	17

**Table 5:** Finite element prediction of the effect of different roller speeds on titanium chip crushing (18 teeth).

Knife Roller Speed/rpm	Length of Titanium Chip After Breaking/mm						
18	5.5	27	31	6	-	-	-
22	13	32	27.5	21.5	-	-	-
26	6.5	25	27	28.5	-	-	-
30	24	28	26	27	10	-	-

**Table 6:** Finite element prediction of the effect of different roller speeds on titanium chip crushing (23 teeth).

Knife Roller Speed/rpm	Length of Titanium Chip After Breaking/mm						
18	23.5	29.5	30	-	-	-	-
22	4.5	27	29	27	-	-	-
26	0.5	33	28	31	27	-	-
30	0.5	25	30	31	27	-	-

**Table 7:** Finite element prediction of the effect of different roller speeds on titanium chip crushing (27 teeth).

Knife Roller Speed/rpm	Length of Titanium Chip After Breaking/mm						
18	9	18.5	19.5	19.5	-	-	-
22	18.5	19	18.5	19.5	18.5	17.5	-
26	18.5	19.5	19.5	19	18	14.5	-
30	1.5	18.5	18	17.5	18	19.5	16.5

**Table 8:** Finite element prediction of the effect of different roller speeds on titanium chip crushing (32 teeth).

Knife Roller Speed/rpm	Length of Titanium Chip After Breaking/mm							
18	5	15.5	14.5	17	16.5	-	-	-
22	2	16.5	15.5	16	15.5	16	-	-
26	1.5	16.5	16.5	16	16	16	17	8
30	1.5	16	15.5	17	16.5	16	17	7.5

**Table 9:** Test design scheme.

Numbering	Experimental Factors			Response Target
	*A*: Teeth Number	*B*: Revolution Speed/rpm	*C*: Angle/°	*f*: Average Length of Titanium Chips/mm
1	26	30	90	17.8
2	22	22	75	27
3	30	30	75	16.3
4	26	26	75	18.2
5	30	26	60	17
6	26	26	75	18.2
7	30	26	90	16.3
8	26	22	60	18.6
9	30	22	75	15.9
10	26	26	75	18.2
11	26	30	60	18.1
12	26	22	90	18.4
13	22	30	75	26.3
14	26	26	75	18.2
15	26	26	75	18.2
16	22	26	60	27
17	22	26	90	26.8

**Table 10:** Variance analysis of titanium chip length.

Type	Sum of Squares	Degree of Freedom	Mean Square Deviation	F	*p*	
Model	163.92	9	18.21	35.29	<0.0001	Significant
A	148.78	1	148.78	288.30	<0.0001	-
B	2.65	1	2.65	0.61	0.44	-
C	3.78	1	3.78	7.33	0.0303	-
AB	1	1	1	1.94	0.2065	-
AC	1.56	1	1.56	3.03	0.1254	-
BC	0.25	1	0.25	0.48	0.5089	-
A²	5.69	1	5.69	11.03	0.0128	-
B²	0.35	1	0.35	0.67	0.4386	-
C²	1.85	1	1.85	3.58	0.1003	-
Residual error	3.61	7	0.52	-	-	-
Misfit term	0.31	3	0.11	0.13	0.9397	Non-significant
Pure differences	3.3	4	0.82	-	-	-
Summation	167.53	16	-	-	-	-

## Data Availability

The original contributions presented in this study are included in this article. Further inquiries can be directed to the corresponding author.
